# Combined hepatocellular carcinoma and cholangiocarcinoma (biphenotypic) tumors: clinical characteristics, imaging features of contrast-enhanced ultrasound and computed tomography

**DOI:** 10.1186/s12885-016-2156-x

**Published:** 2016-02-25

**Authors:** Rui Li, Dan Yang, Chun-Lin Tang, Ping Cai, Kuan-sheng Ma, Shi-Yi Ding, Xiao-Hang Zhang, De-Yu Guo, Xiao-Chu Yan

**Affiliations:** Department Hepato-biliary-Pancreatic Surgery, Southwest Hospital, Gaotangyan street, Shapingba, Chongqing 400038 P. R China; Department Ultrasound, Southwest Hospital Affiliated to Third Military Medical University, Shapingba, Chongqing 400038 P. R China; Department Radiology, Southwest Hospital Affiliated to Third Military Medical University, Shapingba, Chongqing 400038 P. R China; Department Pathology, Southwest Hospital Affiliated to Third Military Medical University, Shapingba, Chongqing 400038 P. R China

**Keywords:** Combined hepatocellular-cholangiocarcinoma, Contrast-enhanced ultrasound, Computed tomography, Alpha-fetoprotein, Carbohydrate antigen 19–9

## Abstract

**Background:**

Combined hepatocellular-cholangiocarcinoma (cHCC-CC) is an uncommon primary liver malignancy and little known about the clinical and imaging characteristics of cHCC-CC. We aim to define the demographics, imaging features of cHCC-CC on contrast-enhanced ultrasound (CEUS) and contrast-enhanced computed tomography (CT) in this study.

**Methods:**

From January 2005 to December 2014, 45 patients with pathologically proven cHCC-CC who underwent preoperative CEUS and 43 patients who had additional CT scan in our institution were included. A retrospective review of the imaging studies and clinical data in these patients was conducted.

**Results:**

In our series, cHCC-CC accounted for 1.6 % of all primary liver malignancy. Mean age of patient with cHCC-CC was 52.8 year (range: 28–74 year) and 88.9 % (40/45) of patients were male. Thirty of forty five patients (66.7 %) had cirrhosis and 20 % (9/45) of patients had chronic hepatitis B without cirrhosis. Alpha--fetoprotein (AFP) was elevated in 62.2 % (28/45) of patients and carbohydrate antigen 19–9 (CA19-9) elevated in 22.2 % (10/45) of patients). Both AFP and CA19-9 were simultaneously elevated in 15.6 % (7/45) of patients. Enhancement pattern resembling cholangiocarcinoma (CC) was noted in 53.3 % (24/45) of patients (on CEUS and in 30.2 % (13/43) of patients at CT. Enhancement pattern resembling hepatocellular carcinoma (HCC) was observed in 42.2 % (19/45) of patients on CEUS and in 58.1 % (25/43) of patients at CT. The percentage of tumors showing CC enhancement pattern (27.9 %, 12/43) was comparable with that of tumors showing HCC enhancement pattern (44.2 %, 19/43) on both CEUS and CT (*p* = 0.116).

Simultaneous elevation of tumor markers (AFP and CA19-9) or tumor marker elevation (AFP or CA19-9) in discordance with enhancement pattern on CEUS was demonstrated in 51.1 % (23/45) of patients and on CT in 53.5 % (23/43) of patients, which was significantly more than simultaneous elevation of tumor markers (AFP and CA19-9) alone (*p* = 0.000).

**Conclusions:**

The clinical characteristics of cHCC-CC are similar to those of HCC. The cHCC-CC tumors display enhancement patterns resembling CC or HCC in comparable proportion on both CEUS and CT. Combination of simultaneous elevation of tumor makers (AFP and CA19-9) and tumor mark elevation (AFP or CA19-9) in discordance with presumptive imaging findings on CEUS or CT may lead significantly more patients to be suspicious of the diagnosis of cHCC-CC.

## Background

Combined hepatocelluar - cholangiocarcinomas (cHCC-CC) are uncommon form of primary hepatic carcinoma, accounts for 1.0–6.3 % of all primary liver cancers in Asia and 2.4–14.2 % of all primary liver cancers in Western countries [1, 2]. It was first defined by Allen and Lisa [3] and has been divided into three types of cHCC–CC. Type A is termed “double cancer” and represents cases in which hepatocelluar carcinoma (HCC) and cholangiocarcinomas (CC) exist separately. Type B, which is called the combined type, is defined as cases in which HCC and CC locates contiguously but independently. The type C, which is referred to as the mixed type, occurs when HCC and CC components intermingle within the same tumor. Only Allen and Lisa type C tumors involved a mixture of both hepatocellular and biliary epithelial cell differentiation, appear to be true biphenotypic cHCC-CC. It is postulated that evolution of cHCC–CC tumors from common hepatic progenitor cells may account for the biphenotypic features of these tumors [4–8]. Due to the relative rarity of this tumor type, little is known about the risk factors, imaging appearance, or prognosis. Few studies have demonstrated risk factors that overlap with hepatocellular carcinoma (HCC) and cholangiocarcinoma (CC), though not all appear to arise in the background of cirrhosis [9]. The clinical characteristics of cHCC-CC were similar to those of HCC [10], but overall survival was more similar to or poorer than that of CC [2, 10]. Survival may be related to tumor biology rather than the cause. Multimodal treatment with an initial aggressive therapeutic approach can improve survival [10]. Given the overlap in patient demographics and the unique nature of cHCC-CC, preoperative diagnosis is crucial for appropriate management. Few studies have evaluated the radiological characteristics of cHCC-CC on computed tomography (CT) or magnetic resonance imaging (MRI) with limited number of patients [11–15]. The presence of imaging features of both HCC and CC in the same tumor may alert the radiologist to the possibility of cHCC-CC though which occurred in the minority of cases [9, 15]. To our knowledge, no study has reported the imaging features of cHCC-CC on contrast-enhanced ultrasound (CEUS) up to now. The main tumor markers of interest are carbohydrate antigen 19–9(CA19-9) and α -fetoprotein (AFP), which are useful adjuncts to imaging in patients with CC and HCC respectively [16]. Simultaneous elevation of both CA 19–9 and AFP has been suggested as highly concerning for cHCC-CC tumors [17]. Other reports suggest that discordance between serum tumor marker elevation and imaging morphology may be suggestive [18]. However, these results were based on clinical data from very limited number of patients and the imaging features of CEUS not included. Therefore, the purpose of this study was to retrospectively evaluate the demographics, clinical presentation, and imaging features on CT and CEUS in patients with cHCC-CC tumors, in the hope of defining features of the uncommon malignant hepatic tumor that may improve preoperative diagnosis and better guide clinical management decisions.

## Methods

### Patient population and clinical information

Institutional review board approval for this study was obtained from the ethics committee of Southwest hospital. The need for informed consent of patient was waived by the ethics committee of Southwest hospital in this retrospective study. From January 2005 to December 2014, pathology databases of our hospital recorded 2863 patients with primary liver cancer, including 46 patients with mixed type (biphenotypic) of cHCC-CC which accounted for 1.6 % of all primary liver cancer. One case of cHCC-CC without available CEUS imaging was excluded from the present study. Patients with available cross sectional imaging were included in the study. Clinical information was retrospectively found from our hospital information system. Serum tumor markers reported were drawn before treatment and within 1 week of the imaging examination. Normal values were 0–20 ng/ml for AFP and 0-22U/ml for CA19-9 in our hospital. Cirrhosis was confirmed histopathologically through examination of resected liver specimen.

All the 45 tumors were excised and underwent tissue diagnosis of the surgical specimen. Liver sections were examined by pathologists with over 20 year experience of liver pathology (XCY and DYG). The final diagnosis of biphenotypic primary liver carcinoma (both types of tumor intermixed) depended on a combination of H and E stain findings [19] and proof of both hepatocellular (polyclonal arcinoembryonic antigen, a marker of canalicular formation,) and biliary differentiation (keratin 7 or keratin19) immunohistochemical markers. Sub-classification was done according o the 2010 World Health Organization Tumors of the Digestive System classification [20]. In patient with more than one lesion,the pathological diagnosis of the largest one was confirmed biphenotypic cHCC-CC. Another 3 patients with double cancer (HCC and CC exist separately) and 7 patients with combined tumors (both types of tumor contiguous to each other) were excluded from the present study.

We performed a per-patient analysis. In a patient with more than one solid lesion in the liver, only the largest one was measured and investigated because CEUS could not scan multiple nodules simultaneously after one injection of contrast agent if the nodules are not at the same scan plane and it is difficult to correspond the pathology of each tumor to the imaging of US in patients with multiple hepatic lesions.

### US scan

US examinations were performed by two experienced physicians with over 16 years of experience of liver ultrasound examination (R L, and XH Z) with an Acuson Sequoia 512 ultrasound unit (Siemens Medical Solutions, Santa Clara, Calif). Baseline US was performed with a multifrequency 4C1 convex array probe. The gray-scale US characteristics of the lesion, including location, size, shape, and echogenicity were recorded. CEUS was performed by using contrast pulse sequencing (CPS) imaging. Real-time contrast imaging setting was used with a low mechanical index of <0.2 and a volume of 2.4 mL of blood pool contrast agent (SonoVue, Bracco Imaging B.V, Geneva, Switzerland) was injected into cubital vein in bolus via a 20-gauge needle followed by a 5 mL saline flush. After contrast medium injection, hepatic lesion was scanned continuously for up to 4 min. The whole vascular phase was studied, consisting of the arterial phase (0–30 s from beginning of contrast agent bolus injection), portal phase (31–120 s after the injection), and delayed phase (121–240 s after the injection) according to EFSUMB recommendations [21]. All ultrasound images of cHCC-CC tumors were reviewed retrospectively for this study by two physicians (R L, and XH Z) in consensus.

### CEUS image analysis

The contrast vascular patterns on CEUS were defined by comparing the enhancement behaviour of the tumor with the surrounding liver parenchyma and classified as:

1. Peripheral hyperenhancement—irregular rim-like hyperenhancement at the peripheral part of the lesion with sparse filiform and punctiform internal enhancement.
2. Heterogeneous hyperenhancement—when the lesion displays mixed hyperenhancement inhomogeneously at both the periphery and the central part of the lesion.
3. Homogeneous hyperenhancement—when the whole lesion shows hyperenhancement homogeneously.
4. Hypoenhancement—the lesion enhances in the less degree than that of the surrounding liver parenchyma.
5. Isoenhancement—the lesion enhances in the similar degree as the surrounding liver parenchyma.
6. Non-enhancement—no appearance of contrast agent (microbubbles) at both the periphery and the central part of the lesion.

Wash-out appearance was considered as the presence of hypoenhacement of the lesion in the portal or late phases preceded by hyperenhancement in the arterial phase. A lesion lacking any enhancement in all phases was not defined as washout. When a nodule shows heterogeneous hyperenhancement in the arterial phase, observation of washout was confined to the area showing hyperenhancement in the arterial phase, and the area showing non-enhancement was excluded from the observation. The extent of washout was classified as:

1. Marked washout—when the lesion displays obviously lower echogenicity than the surrounding liver parenchyma in the portal or late phases preceded by hyperenhancement in the arterial phase.
2. Mild washout —when the lesion shows slightly lower echogenicity than the surrounding liver parenchyma in the portal or late phases preceded by hyperenhancement in the arterial phase.
3. No washout—when the lesion enhances in the similar degree or higher degree as the surrounding liver parenchyma in the portal or late phases preceded by hyperenhancement in the arterial phase.

Typical HCC pattern on CEUS was defined as hyperenhancement in the arterial phase followed by slow or slight washout in the portal or late phase,and the wash out tends to start later in HCC, usually not before 60 s after injection [22, 23]. On the contrary, most peripheral cholangiocarcinoma showed wash out emergence before 60 s and marked wash out in the portal phase on CEUS [24, 25]. Typical CC pattern on CEUS was defined as: (1) Peripheral arterial rim enhancement followed by marked washout in the portal phase; (2) Arterial hyperenhancement followed by quick (washout begins before 60 s after contrast agent injection) and marked washout in the portal phase [22, 24, 25].

### CT scan

Abdomen CT was performed with multidetector-row CT (MDCT, 64 detector rows, Definition, Siemens, Erlangen, Germany) using a 4 phase contras-enhanced protocol (unenhanced, hepatic arterial, portal venous, and delayed phases). First, an unenhanced scan was obtained through the liver. Next, after intravenous infusion of 2 ml /kg of a nonionic iodine-containing contrast agent (ultravist 370, Scherning AG, Berlin, Germany) using a power injector (Stellant CT Injection System, Medrad, Indianola, Pennsylvania) at a rate of 4.0 ml/s, contrast-enhanced scans were obtained in arterial with bolus test trigger for optimal characterization of focal hepatic lesions. Data acquisitions were obtained through the whole liver in a craniocaudal direction during a single breath-hold helical acquisition for 4–6 s with 5 mm slice thickness and 0.5 s rotation time. The acquisition of the arterial phase was automatically started 5 s after contrast agent reaching the threshold in the aorta. The start of acquisition sequences was 60 s for the portal venous phase and 180 s for the delayed phase.

### Categorization of enhancement patterns at CT

The enhancement through each of the different phases after intravenous contrast administration was registered as follows: (1) globally hyperdense: increased signal relative to the surrounding liver parenchyma, involving the totality of the lesion; (2) partially hyperdense: increased signal involving more than 50 % of the lesion cross-section area with a non-homogeneous distribution; (3) peripherally hyperdense: increased signal limited to the periphery of the lesion, involving less than 25 % of its area, resembling a rim-like pattern; (4) isodense: same density as the surrounding liver parenchyma; (5) hypodense: lower density compared to the liver parenchyma involving more than half of the cross-sectional area of the tumor. Dynamic pattern of enhancement was defined according to the analysis of the progression of contrast enhancement over different phases of the study, as follows: (1) stable or persistent contrast enhancement: the nodule enhancement is unmodified from the arterial to the portal venous and delayed phases; (2) progressive contrast enhancement: the nodule enhances progressively over time, reaching maximal intensity in delayed phases; (3) ‘wash-out’ pattern: global intense/partial hyperdense of the lesion during the arterial phase followed by hypodense in portal and/or delayed venous phases; (4) all other cases. This classification was adopted from Rimola et al. [26] and lavarone et al. [27].

CT findings were evaluated in consensus by 2 abdominal radiologists (PC and SYD) with over 15 years of experience in liver radiology who were blinded to CEUS findings and pathological results of the tumors. Typical HCC pattern on CT was defined as hyperenhancement in the arterial phase followed by washout in the portal or late phase of the nodule [28]. Typical CC pattern on CT was defined as progressive centripetal enhancement or stable persistent enhancement of the nodule [27].

### Statistical analysis

We performed a per-patient analysis in this study. Characteristics of the patients are expressed as median and range or count and proportion. Comparison of CEUS and CT was done by using the chi-squared test for categorical variables. A *P* value of less than 0.05 was considered statistically significant. Statistical analysis was performed using the SPSS 13.0 software package (SPSS Inc, Chicago, IL).

## Results

### Clinicopathologic features

Demographics of 45 patients with cHCC-CC are shown in Table 1. Mean age of patients was 52.8 year (range: 28–74 year) and 40 patients were male (88.9 %). Thirty patients (66.7 %) had cirrhosis. The etiology of cirrhosis was viral hepatitis B infection in 24 patients, combination of viral hepatitis B infection and alcohol misuse in 5 patients. Nine patients had chronic hepatitis B without cirrhosis (20 %). Of the 45 patients with cHCC-CC tumors, 6 presented incidentally, 10 discovered on cirrhosis screening, 22 presented with abdominal pain, 2 with jaundice, 2 with tarry stool, 1 with edema of lower limbs. Of the 22 patients presented with abdominal pain, 4 patients had a palpable mass. In two patients, the presentation is unknown. All the 45 patients had AFP assay which was abnormally elevated in 28 patients (62.2 %) and normal in 17 patients. Forty three patients had serum assays of carcinoembryonic antigen (CEA) which was elevated in 5 patients (11.6 %) and normal in 38 patients. Forty two patients had serum assays of CA-125 which was elevated in 6 patients (14.3 %) and normal in 36 patients. Forty five patients had serum assays of CA19-9 which was elevated in 10 patients (22.2 %) and normal in 35 patients. Both AFP and CA19-9 were simultaneously elevated in 7 patients (15.6 %). The average size of cHCC-CC was 5.3 cm and over half of them were less than 5 cm. Most patients had single tumor and over half of them were located in the right lobe of liver. In 6 patients with 2 hepatic nodules, pathological diagnosis of all the nodules was cHCC-CC. In 1 patient with 4 hepatic nodules, pathological diagnosis of 3 nodules, including the largest one was cHCC-CC, and 1noule was heamangioma.

**Table 1:** Demographics of 45 patients with combined hepatocellular-cholangiocarcinoma

	Number (%)	Mean ± SD	Median (range)
Age (year)		52.8 ± 10.4	51.5 (28–74)
Male/female	40/5		
AFP (ng/ml)		680.8 ± 1700.2	68.6 (1.02–9675)
≤ 20	17 (37.8)		
21–200	15 (33.3)		
> 200	13 (28.9)		
Underling liver disease			
Cirrhosis	30 (66.7)		
Chronic hepatitis B	4 (8.9)		
Chronic hepatitis C			
Alcoholic	3 (6.7)		
Chronic hepatitis B and alcoholic	5 (11.1)		
No evidence of chronic liver disease	3 (6.7)		
Nodule size (cm)		5.3 ± 3.2	4.5 (1.5–13.8)
< 5 cm	24 (53.3)		
5–10 cm	15 (33.3)		
> 10 cm	6 (13.3)		
Number of nodules			
Single	38 (84.4)		
Two	6 (13.3)		
More than three	1 (2.2)		
Location of nodules			
Left lobe	8 (17.8)		
Right lobe	31 (68.9)		
Both left and right lobe	3 (6.7)		
Caudate lobe	3 (6.7)		
Echogenicity of nodules			
Hypoechoic	23 (51.1)		
Isoechoic	2 (4.4)		
Hyperechoic	12 (26.7)		
Mixed	8 (17.8)		

### Radiological features

The enhancement appearances of cHCC-CC on CEUS in 45 patients are shown in Table 2. Tumors showing peripheral hyperenhancement in the arterial phase followed by marked washout in the portal phase in 9 patients were defined as CC pattern. Tumors showing heterogeneous (8 tumors) or homogeneous (7 tumors) hyperenhancement in the arterial phase followed by early (washout begins earlier than 60 s) and marked wash out during the portal phase were judged as CC pattern. Tumors showing heterogeneous (13 tumors) or homogeneous (6 tumors) hyperenhancement in the arterial phase followed by both slow (washout begins later than 60 s) and mild wash out in the portal or late phase were judged as HCC pattern. The enhancement pattern of 2 patients was judged as indeterminate because one tumor showed heterogeneous hyperenhancemen in the arterial phase followed by isoenhancement in the portal and the late phase, and another tumor displayed hypoenhancement in the arterial phase, remained hypoenhancement in the portal and the late phase. The average time of washout emergence on CEUS was 58.18 s (median :53 s, range :22 s ~ 129 s). The percentage of tumors showing CC enhancement pattern (53.3 %) was similar to that of tumors showing HCC enhancement pattern (42.2 %) on CEUS (*p* = 0.291).

**Table 2:** The enhancement patterns of combined hepatocellular-cholangiocarcinoma on CEUS in 45 patients

Enhancement appearance	Number %
Arterial phase	
Peripheral hyperenhancement	9 (20.0)
Heterogeneous hyperenhancemen	22 (48.9)
Homogeneous hyperenhancement	13 (28.9)
Hypoenhancement	1 (0.2)
Isoenhancement	0 (0)
Portal phase	
Peripheral hyperenhancement	
Heterogeneous hyperenhancement	1 (0.2)
Homogeneous hyperenhancement	0 (0)
Slight hypoenhancement	17 (37.8)
Marked hypoenhancement	25 (55.6)
Isoenhancement	2 (0.4)
Delayed phase	
Peripheral hyperenhancement	
Heterogeneous hyperenhancement	1 (0.2)
Homogeneous hyperenhancement	
Slight hypoenhancement	8 (17.8)
Marked hypoenhancement	35 (77.8)
Isoenhancement	1 (0.2)
Emergence of-washout	
< 60 seconds	28 (62.2)
60–120 seconds	13 (28.9)
> 120 seconds	2 (4.4)
No washout	2 (4.4)

Forty three patients had unenhanced CT scan. Forty of the tumors were hypodense and 3 were hyperdense. Hepatic capsular retraction was revealed in 4 cases (9.3 %). Three patients had intrahepatic biliary dilatation (7.0 %). Five patients had malignant portal veins thrombus (11.6 %) and 5 had regional lymphadenopathy (11.6 %). Intrahepatic metastasis was observed in 7 patients (16.3 %). Enhancement appearances of cHCC-CC on contrast-enhanced CT in 43 patients are shown in Table 3. Tumors showing stable persistent peripherally hyperenhancement from the arterial phase to the late phase in 8 patients were defined as CC pattern. Tumors showing progressive delayed enhancement from the arterial phase to the late phase in 5 patients were also judged as CC pattern. Tumors showing hyperenhancement (24 heterogeneous, 1 homogeneous) in the arterial phase followed by washout in the portal or the late phase in 25 patients were defined as HCC pattern. Tumors showing hypoenhancement from the arterial phase to the late phase in 4 patients and 1 tumor showing hyperenhancement in the arterial phase and the portal phase followed by isoenhancement in the late phase were judged as indeterminate pattern. The percentage of tumors showing CC enhancement pattern (30.2 %) was less than that of tumors showing HCC enhancement pattern (58.1 %) on CT (*p* = 0.009). Twelve of forty three patients displayed CC enhancement pattern on both CEUS and CT (27.9 %), while 44.2 % (19/43) of patients demonstrated HCC enhancement pattern at both CEUS and CT. The percentage of tumors showing CC enhancement pattern was comparable with that of tumors showing HCC enhancement pattern (*p* = 0.116).

**Table 3:** Enhancement patterns of combined hepatocellular-cholangiocarcinoma on CT in 43 patients

	Arterial phase	Portal phase	Delayed phase
Peripherally hyperdense No.(%)	12 (27.9)	13 (30.2)	7 (16.3)
Partially hyperdense No.(%)	24 (55.8)	5 (11.6)	6 (13.9)
Globally hyperdense No.(%)	2 (0.5)		
Isodense No.(%)			1 (0.2)
Hypodense No.(%)	5 (11.6)	25 (58.1)	29 (67.4)

### Correlations of AFP and CA19-9 levels with enhancement patterns on CEUS and CT

**Fig. 1:**
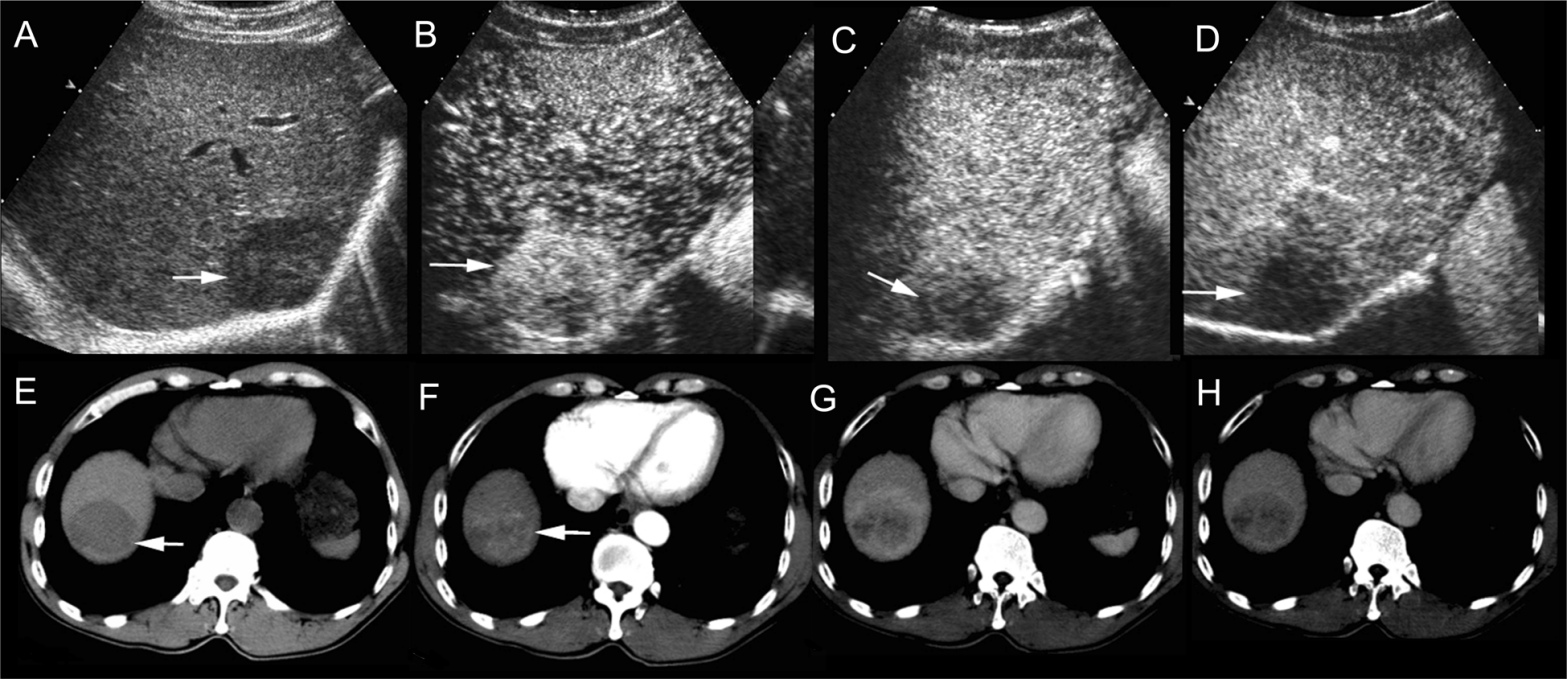
cHCC-CC in a patient with chronic hepatitis B. Simultaneous elevation of AFP (718 ng/ml) and CA19-9 (45.0U/ml) was detected in the patient. Unenhanced ultrasound shows a hypoechoic mass of 4.6 cm in right lobe of the liver (**a**, arrow). The mass displays heterogeneous hyperenhancement in the arterial phase (**b**, 26 s after contrast agent injection) followed by quick (**c**, 58 s after injection) and marked washout (**d**, 102 s after injection) in the portal phase on CEUS, resembling the enhancement pattern of intrahepatic cholangiocarcinoma. Unenhanced CT scan reveals a hypodense mass in right lobe of the liver (**e**, arrow). The mass displays heterogeneous hyperenhancemen in the arterial phase (**f**, arrow) followed by washout (**g**) in the portal phase and the late phase (**h**) on contrast-enhanced CT, resembling the enhancement pattern of HCC

**Fig. 2:**
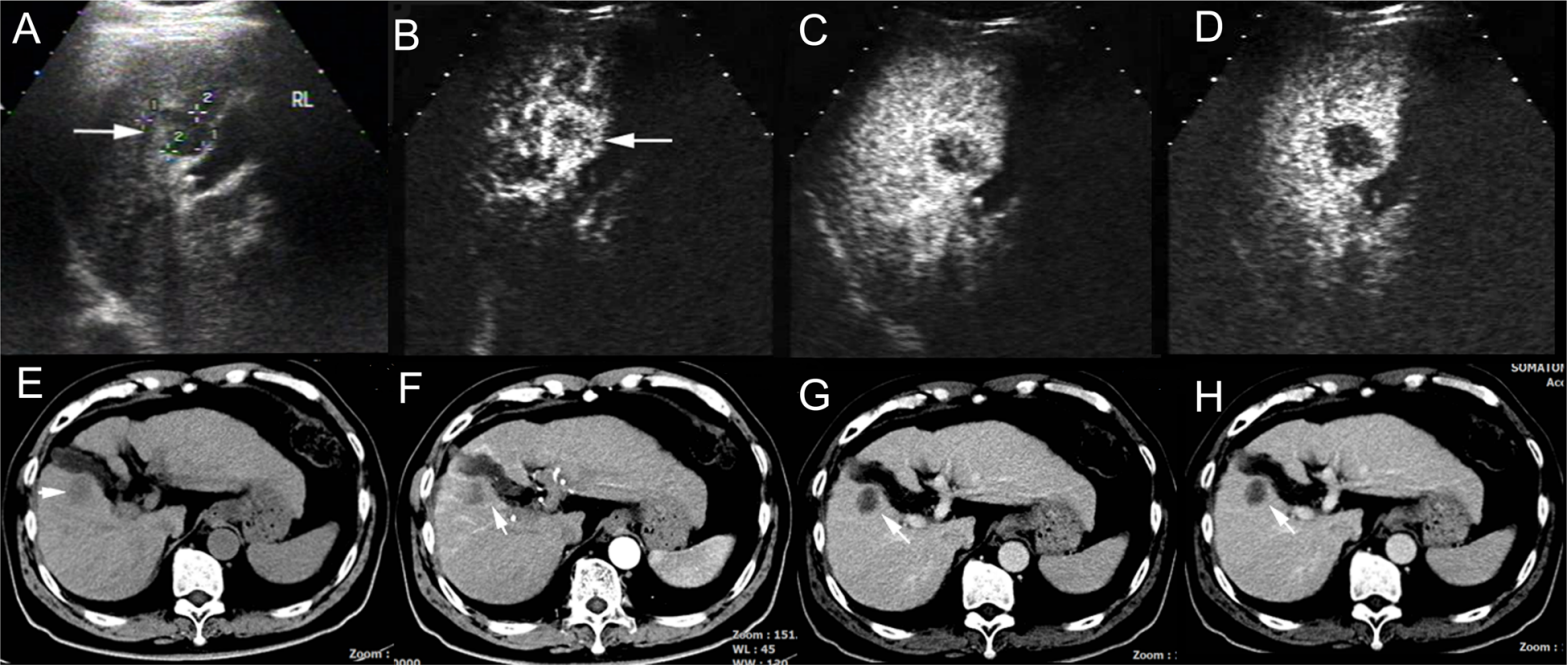
cHCC-CC in a patient with cirrhosis related to chronic hepatitis B infection. CA19-9 test was normal and AFP elevated (178.5 ng/ml) in discordance with presumptive imaging findings in the patient. Unenhanced ultrasound shows a hypoechoic nodule (**a**, arrow) of 2.7 cm in right lobe of the liver (RL). The nodule demonstrates peripheral rim-like enhancement in the arterial phase (**b**, 18 s after contrast agent injection, arrow) followed by quick (**c**, 37 s after injection) and marked washout (**d**, 82 s after injection) in the portal phase on CEUS, resembling the enhancement pattern of intrahepatic cholangiocarcinoma. Unenhanced CT scan reveals a hypodense nodule in right lobe of the liver (**e**, arrow). The nodule displays peripheral rim-like enhancement in the arterial phase (**f**, arrow) followed by stable and persistent peripheral rim-like enhancement in the portal (**g**) and the late phase (**h**, arrow), resembling the enhancement pattern of intrahepatic cholangiocarcinoma

**Fig. 3:**
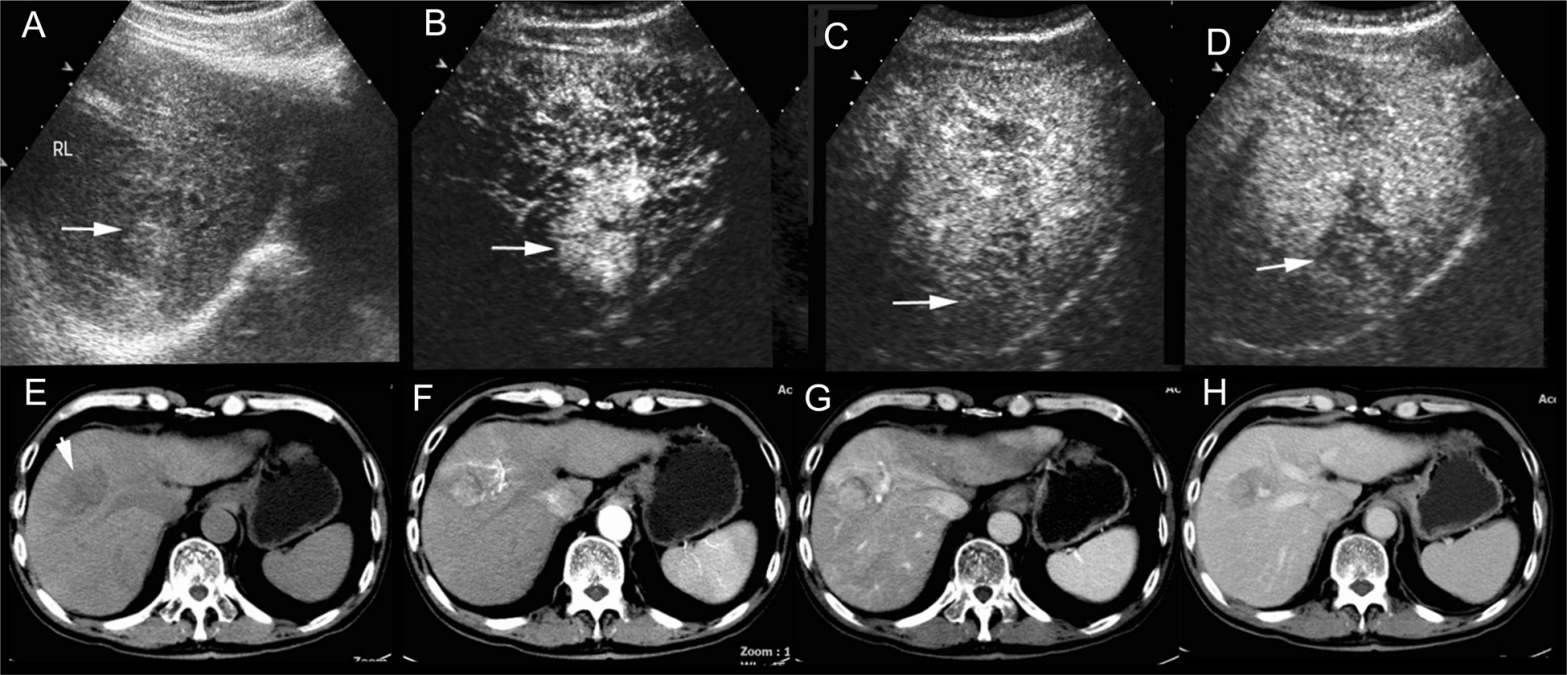
cHCC-CC in a patient with cirrhosis related to chronic hepatitis B infection. AFP test was normal and CA19-9 elevated (50.3U/ml) in discordance with presumptive imaging findings in the patient. Unenhanced ultrasound shows a hyperchoic mass of 3.7 cm with hypoechoic halo (arrow) in right lobe of the liver (**a**). The mass displays heterogeneous hyperenhancemen in the arterial phase (**b**, 29 s after contrast agent injection) followed by slow washout in the portal phase (**c**, 110 s after injection, arrow) and mild washout (**d**, 179 s after injection, arrow) in the late phase on CEUS, resembling the enhancement pattern of HCC. Unenhanced CT scan reveals a hypodense mass in right lobe of the liver (**e**, arrow). The mass displays heterogeneous hyperenhancement in the arterial phase (**f**) and the portal phase (**g**) followed by washout in the late phase (**h**) on contrast-enhanced CT, resembling the enhancement pattern of HCC

In 7 patients with simultaneous elevation of both AFP and CA19-9, CC enhancement pattern was observed in 5 patients on CEUS and 1 patient at CT. HCC enhancement pattern was noted in 2 patients on CEUS and 6 patients at CT respectively (Fig. 1). Correlations between AFP test and enhancement patterns of CEUS and CT are shown in Table 4. AFP was elevated and CA19-9 normal in 9 of 45 (20.0 %) patients showing CC enhancement pattern on CEUS and in 7of 43 (16.3 %) patients showing CC enhancement pattern on CT (*p* = 0.651) (Fig. 2). In 3 patients with elevated CA19-9 and normal AFP, HCC enhancement pattern was noted in 3 patients on CEUS and in 2 patients at CT (Fig. 3), CC enhancement pattern was observed 1 patient at CT. Correlations between CA19-9 test and enhancement patterns of CEUS and CT are shown in Table 5. Elevated tumor markers (AFP or CA19-9) were in discordance with imaging findings in 19 of 45 (42.2 %) patients on CEUS and in 16 of 43 (37.2 %) patients at CT (*p* = 0. 0.631). Simultaneous elevation of tumor markers (AFP and CA19-9) or tumor marker elevation (AFP or CA19-9) in discordance with enhancement pattern on CEUS was demonstrated in 26 of 45 patients, which was significantly more than simultaneous elevation of tumor markers (AFP and CA19-9) alone (7/45, *p* = 0.000). Simultaneous elevation of tumor markers (AFP and CA19-9) or tumor marker elevation (AFP or CA19-9) in discordance with enhancement pattern on CT was observed in 23 of 43 patients, which was significantly more than simultaneous elevation of tumor markers (AFP and CA19-9) alone (7/43, *p* = 0.000).

**Table 4:** Correlation between AFP test and enhancement patterns of CEUS and CT

	CEUS pattern (n = 45)			CT pattern (n = 43)		
	CC	HCC	indeterminate	CC	HCC	indeterminate
AFP normal	10	7		5	9	3
AFP elevated	14	12	2	8	16	2

**Table 5:** Correlation between CA19-9 test and enhancement patterns of CEUS and CT

	CEUS pattern (n = 45)			CT pattern (n = 43)		
	CC	HCC	indeterminate	CC	HCC	indeterminate
CA19-9 normal	19	14	2	11	17	5
CA19-9 elevated	5	5		2	8	

## Discussion

Combined hepatocellular-cholangiocarcinoma accounts for 0.4–14.2 % of all primary liver carcinomas, with not only local incidence varying considerably between regions [16, 18], but also the different including criteria for classification in previous literature. Although some published reports involving only Allen and Lisa type C of cHCC-CC [18, 29], others either included all three types of cHCC-CC [30, 31] or did not state their criteria for diagnosis and classification [32, 33]. From January 2005 to December 2014, 46 out of 2863 patients underwent surgery or biopsy for hepatic malignancy at our hospital had unequivocal mixed type (biphenotypic) of cHCC-CC. The incidence of this real mixed type cHCC-CC composed of cholangiocyte-derived and hepatocyte-derived neoplastic elements is 1.6 %. In the present series, most patients with cHCC-CC had cirrhosis (66.7 %) or chronic hepatitis B (20 %). The mean age of patients was 52.8 year and 88.9 % of patients were male. Our data demonstrated that cHCC-CC developed more frequently in a middle-aged male population with chronic hepatitis and cirrhosis mostly related to chronic hepatitis B, indicating the clinical characteristics of cHCC-CC are similar to those of HCC [10, 34]. However, this is inconsistent with two reports from Western countries [15, 18], which published data of 27 patients and 29 patients, cirrhosis was seen in 0 % and 20 %, positive hepatitis B or C detected in 15 % and 10 % of the patients respectively. The discrepancy may relate to different population background characteristics, reflecting the high rates of hepatitis B viral infection in Chinese populations. Although the majority of patients had insidious onset of cHCC-CC with late presentation of advanced disease, 35.6 % of our patients were discovered on cirrhosis screening or presented incidentally at early stage for regular health examination by ultrasound.

In our study, 27.9 % of the cases showed arterial peripheral hyperenhancement on CT, which is lower than previous reports by Ebied et al. (50 %) and Fowler et al. (51.9 %) [13, 15]. On the contrary, 60.5 % of our patients displayed heterogeneous or homogeneous hyperenhancement in the arterial phase that is higher than in the study by Ebied et al. (33.3 %). Patients with cHCC-CC in the present study demonstrated more HCC enhancement pattern (58.1 %) and less CC enhancement pattern (30.2 %) than reported by Fowler et al. (31.0 %, 41.4 % respectively). An explanation of these discrepancies may be the tumor size, which was much smaller in our study (median size 4.5 cm) as compared with the data reported by Ebied et al. (median size 7 cm) and by Fowler et al. (median size 7.5 cm).As the tumor grows larger, a relatively smaller blood supply is available, leading to necrosis and more fibrous stroma formation in the central portion of the tumor, which constitute the pathological background of CC enhancement pattern on contrast-enhanced CT [35]. Capsular retraction and biliary ductal dilatation have been considered important ancillary features of CC. Less patients with cHCC-CC revealed capsular retraction (9.3 %) and biliary ductal dilatation (7.0 %) on CT in our data than reported by Ebied et al. (26.7 %, 16.7 %) and by Fowler et al. (41.4 %, 34.5 %). We favor to interpret these inconformity in the light of the differences in tumor size and the underling liver diseases, namely, the tumors of our patients were much smaller and more patients had cirrhosis than in the previous series mentioned above.

Imaging characteristics of cHCC-CC on CEUS has not been reported up to now. Our study demonstrated that 95.6 % of the tumor showed washout enhancement pattern on CEUS, indicating a malignant nature of the tumor. Imaging features of cHCC-CC may display as CC enhancement pattern or HCC enhancement pattern. The percentage of the two types of enhancement pattern on CEUS showed no statistical difference in our series. Our results demonstrated that imaging features of cHCC-CC may resemble either CC or HCC.CC enhancement pattern and HCC enhancement pattern are likely present in a comparable proportion in patients with cHCC-CC on CEUS.

Serum tumor markers of potential utility in cHCC-CC are CA 19–9 and AFP, which are associated with CC and HCC respectively. When both are simultaneously elevated or elevated in discordance with presumptive imaging findings (i.e., elevated CA 19–9 with imaging findings of HCC pattern, or elevated AFP with imaging findings of CC pattern), cHCC-CC should at least be suggested [9, 17]. However, this point of view was based on few studies with very limited number of patients (less than 15 patients) [11, 17, 36] and needs to be evaluated in more patients. In addition, the imaging features of cHCC-CC on CEUS have not been taken into consideration up to now. Previous reports demonstrated that elevated serum AFP levels were found in 33 %–78 % and elevated CA19-9 in 20 %–36 % of patients with cHCC-CC [11, 17, 36]. In our series, AFP was abnormally elevated in 62.2 % and CA19-9 in 22.2 % of patients, which is comparable to previous reports. AFP and CA19-9 were simultaneously elevated in 15.6 % of patients in the present study, indicating a much low sensibility if this criterion alone was used for suggestion of cHCC-CC. In our study, simultaneous elevation of tumor markers (AFP and CA19-9) or tumor marker elevation (AFP or CA19-9) in discordance with enhancement pattern on CEUS or on CT was demonstrated in significantly more patients (51.1 %, 53.3 % respectively) than simultaneous elevation of tumor markers (AFP and CA19-9) alone (15.6 %, *p* = 0.000), indicating that when both the results of tumor makers and imaging features of CEUS or CT were taken into consideration, the possibility of cHCC-CC may be suggested in significantly more patients.

There are some limitations in the present study. First, it is a retrospective analysis, though the rarity of cHCC-CC lends itself almost exclusively retrospective design. Second, the patient population is relatively small, consisting of 45 patients with cHCC-CC out of 2863 cases of primary liver malignancy. Nevertheless, our sample size is relatively larger than most previously published series. Third, only 2 patients in our study had MRI examination before operation, the imaging features of cHCC-CC on MRI could not be summarized. This reflects the fact that in many countries worldwide, with a high incidence of HCC, the availability of MRI scan for the diagnosis of focal liver lesions is still much low.

## Conclusions

Combined hepatocelluar – cholangiocarcinomas (biphenotypic) is an uncommon primary liver malignancy with background population characteristics similar to HCC. Imaging features of CC or HCC presents in comparable proportion in cHCC-CC on both CEUS and CT. Combination of simultaneous elevation of tumor makers (AFP and CA19-9) and tumor mark elevation in discordance with presumptive imaging findings on CEUS or CT may lead significantly more patients to be suspicious of the diagnosis of cHCC-CC.

## Abbreviations

cHCC-CC: Combined hepatocelluar – cholangiocarcinomas
HCC: Hepatocelluar carcinoma
CC: Cholangiocarcinomas
CT: Computed tomography
MRI: Magnetic resonance imaging
CEUS: Contrast-enhanced ultrasound
CA19-9: Carbohydrate antigen 19–9
AFP: α –fetoprotein
